# 

**Published:** 1917-06-09

**Authors:** 


### CONTENTS.

| | PAGE |
|---|---|
| **The Prevention of Venereal Disease** ... ... | 179 |
| **The War and the Future of Poor-Law Infirmaries** ... ... ... ... ... | 180 |
| **Hospital and Institutional News** ... ... ... | 181 |
| British Ambulances with the French Army—The Scarcity of Doctors—The Fruits of THE HOSPITAL'S Teaching—The Bartletts and East Suffolk Hospital—A Laughable Confession—Presentation to a Doctor—Mrs. Lloyd George at the Swansea Infirmary—The Aldershot Workers' Hospital Fund—Hospital as a "Mobilisation Centre"—The Red Cross "Victory" Sale—Red Cross Work in Sussex—The Lord Mayor at Dollis Hill—Another Building Fund—Hospital Extension at Nottingham—The West Kent Hospital Pay Day Fund—A War Council Concession—A Motor Laboratory for the French Red Cross—The Effective Use of Radium—A Soldier's Tribute to Homœopathy—Is a Health Visitor a Luxury?—This Week's Drug Market—To Our Readers. | |
| **Milk for Infants at Schools for Mothers** ... | 185 |
| The National Clean Milk Society's Investigation. | |
| **The Venereal Clinics of the Seamen's Hospital Society** ... ... ... ... ... | 186 |
| **A "Hut" Hospital** ... ... ... ... ... | 187 |
| How the 3rd London General Hospital came into being. | |
| **Birthday Honours** ... ... ... ... ... ... | 189 |
| **The Editor's Letter-Box** ... ... ... ... | 190 |
| The Case Against the Voluntary Hospital. | |
| **The Matrons' and Sisters' Department** ... | 191 |
| The Preliminary Training School for Nurses: III. The Week's Work. | |
| Round the Hospitals. | |
| The Central Committee's Position: Has it Forfeited its Legal Status?—State Registration in Scotland—Condemnation of Central Committee's Action—Refusal to Sign any Petition—College Speakers at Manchester—Birmingham Guardians and Nurses' Salaries—Comforts for Institution Workers—Royal Gwent Hospital, Newport, Mon. | |
| **Food in Wartime** ... ... ... ... ... | 194 |
| A Kitchen Committee. | |
| **Mothercraft and Child Welfare** ... ... ... | 195 |
| Maternity as State Service. | |
| **The London Hospital** ... ... ... ... ... | 196 |
| Quarterly Report of the House Committee. | |
| **Matrons' and Sisters' Appointments** ... ... | iv |
| **The Book Market** ... ... ... ... ... ... | iv |
| **The Institutional Worker** ... ... (Suppl.) | 1 |
| Answer to April Question. | |

